# Is collaborative care a key component for treating pregnant women with psychiatric symptoms (and additional psychosocial problems)? A systematic review

**DOI:** 10.1007/s00737-022-01251-7

**Published:** 2022-09-26

**Authors:** Celine K. Klatter, Leontien M. van Ravesteyn, Jelle Stekelenburg

**Affiliations:** 1 Department of Global Health, Medical Sciences, University of Groningen/University Medical Centre Groningen, Hanzeplein 1, 9713 GZ Groningen, The Netherlands; 2 Department of Obstetrics and Gynaecology, Medical Centre Leeuwarden, Henri Dunantweg 2, 8934 AD Leeuwarden, The Netherlands

**Keywords:** Pregnancy, Mental disorders, Psychosocial, Collaborative care

## Abstract

Mental disorders during pregnancy are common, with long-lasting negative effects on mother and child. Treatment of these women is challenging, because of the high incidence of additional psychosocial problems and barriers on population and healthcare level. Collaborative care, collaboration between mental health and obstetric care professionals, may help to overcome these problems. The aim of this review is to review antenatal mental health interventions and analyse the impact of collaborative care. Two independent reviewers searched for RCT’s in PubMed, Embase and PsycINFO. Trials studying the effect of psychological or pharmacological interventions on the mental health of pregnant women with psychiatric symptoms (and psychosocial problems) were eligible for inclusion. Two reviewers independently abstracted data and assessed study quality and risk of bias. Each study was scored on collaborative care criteria: multi-professional approach to patient care, structured management plan, scheduled patient follow-ups and enhanced interprofessional communication. Thirty-five studies were included. Most trials studied the effect of cognitive behavioural therapy and interpersonal psychotherapy on antenatal depression. Almost all interventions met at least one collaborative care criteria. Interventions were mostly provided by multiple professionals, but interprofessional communication rarely took place. Interventions that met more criteria did not more often show a positive effect on maternal mental health. There is lack of research on antenatal psychiatric disorders other than depressive and on long-term treatment outcomes. Collaborative care is partly implemented in most current interventions, but more trials (including interprofessional communication) are needed to be conclusive whether collaborative care is a key component in antenatal mental healthcare.

**Supplementary Information:**

The online version contains supplementary material available at 10.1007/s00737-022-01251-7.

## Introduction

Globally, mental disorders during pregnancy are common, with prevalence rates of depressive and anxiety disorders around 20% (Woody et al. 2017; Ross and Dennis 2009, Yin et al. 2021). Women disadvantaged by psychosocial problems (low-income, minority status, unsafe (home) situation, living place in a deprived area, substance use disorder) are even more likely to develop depressive disorders (Ogbo et al. 2018; Redshaw and Henderson 2013). The prevalence rate in this population lies between 5 and 47% (Ross and Dennis 2009, Bennett et al. 2004). Mood and anxiety disorders do not only affect pregnant women by increasing the risk of postpartum depression and psychosis, but also have negative effects on the (unborn) child. These are risk factors for caesarean section, low birthweight and behavioural problems in late childhood (Jarde et al. 2016; Yedid Sion et al. 2016; Lee et al. 2007; Leis et al. 2014). There is growing evidence showing that mental disorders during pregnancy are common and have long-lasting consequences.

Current treatment options for antenatal depressive and anxiety disorders can be broadly divided into psychotherapy and pharmacotherapy. Because antenatal use of medication (e.g. antidepressants and/or anxiolytics) may have adverse effects on the (unborn) child (e.g. transient neonatal symptoms, congenital abnormalities and risk of pre-term birth), clinicians and women often prefer psychotherapy (Pearlstein 2015). Studies show that cognitive behavioural therapy (CBT) and interpersonal psychotherapy (IPT) can be effective in reducing antenatal depressive and anxiety symptoms (Sockol 2015; Sockol 2018). However, these therapies seem less effective for women with additional psychosocial problems (Nillni et al. 2018). Also, there is limited evidence of the long-lasting effects. Several barriers concerning the organization of care are identified on patient (e.g. stigma), provider (e.g. lack of expertise) and healthcare level (e.g. limited access to mental health treatment) (Byatt et al. 2012). Of all pregnant women diagnosed with a mental disorder, less than 10% receive adequate treatment (antidepressants or psychotherapy for > 6 weeks) and less than 5% achieve remission of psychiatric symptoms (Cox et al. 2016). Women with additional psychosocial problems are even less likely to engage in treatment (Grote et al. 2007). These factors make treating pregnant women with mental disorders challenging.

Collaboration between mental healthcare workers and obstetric care professionals may help overcome these challenges. Research has shown that collaborative care in other fields, like primary mental health care, is effective in improving psychiatric symptoms and adherence to treatment (Thota et al. 2012; Archer et al. 2012). Collaborative care in the obstetric care setting is a multidisciplinary treatment approach consisting of obstetricians, midwives, psychiatrists and psychologists (and social workers). (Gunn et al. 2006) designed a framework that identifies key components of collaborative care. Systematic reviews have used this framework in mental healthcare settings. We adapted these criteria to the obstetric care setting, in order to score studied interventions on the following collaborative care criteria:

1. A multi-professional approach to patient care
2. A structured management plan
3. Scheduled patient follow-ups
4. Enhanced interprofessional communication

Whether collaborative care interventions are effective in treating antenatal mental disorders is unknown. The aim of this study is to review antenatal mental health interventions, assess the level of collaboration and describe the impact of the different quality levels, measured by the collaborative care criteria (CCC), on maternal mental health outcomes.

## Materials and methods

This systematic review is carried out according to the PRISMA guidelines.

### Search strategy and study selection

We searched for randomized controlled trials, published in peer-reviewed journals, in the databases PubMed, Embase and PsycINFO. The following search terms, based on a previous review by LvR (Ravesteyn et al. 2017) and on clinical judgement, were used to identify eligible articles (see online resource 1): (1) pregnancy, (2) mental disorders, (3) psychosocial problems, (4) treatment and (5) randomized controlled trials. We used subject headings and free text terms. The search was conducted on 31 January 2020 and updated on 20 December 2021. We exported the results to EndNote and removed duplicates. Two authors (CK, LvR) reviewed the title and abstract for concept validity and excluded abstracts deemed to be irrelevant. In case of disagreement or doubt, a third reviewer was consulted (JS). Based on the selection criteria described below, full text articles were assessed. When there were multiple articles from the same trial, the article with the most complete or relevant outcomes was included.

### Selection criteria

We included randomized controlled trials from database inception through December 20, 2021, if they.

- included pregnant women diagnosed with a mental disorder according to a standardized diagnostic interview based on the DSM or ICD criteria or pregnant women with clinical psychiatric symptoms according to validated mental health questionnaires (e.g. EPDS ≥ 10). Women with and without additional psychosocial problems (low-income, minority status, unsafe (home) situation, living place in a deprived area, substance use disorder) were included.
- studied a psychological or pharmacological treatment and provided face-to-face or over the telephone by one or more professional healthcare workers (e.g. midwife, obstetrician, psychiatrics, psychologist). Psychological interventions provided by a midwife or obstetrician were also included.
- examined any of the following maternal mental health outcomes: psychiatric symptoms (validated questionnaires, e.g. EPDS, STAI), incidence rates, recovery rates or risk reduction of psychiatric disorders. Secondary outcomes are any birth, neonatal, infant or biomarker outcome.

We excluded non-English studies, abstracts, case reports and studies including postpartum women. Also, studies on solely transcranial stimulation, food supplements, eHealth, physical treatment and interventions provided by informal health workers (e.g. lay health worker, peer support) were excluded.

### Data extraction and analysis

The following details of each study were extracted and described in a summary table: first author, country, setting, (number of) participants, (duration of the) intervention, control condition, maternal mental health outcome and retention and compliance rates. The retention rate is calculated as the percentage of women who remained till the latest follow-up and the compliance rate as the percentage of women who attended all sessions of all women allocated to the intervention group. Birth, neonatal and infant outcomes are separately described.

First, the extracted data was analysed to create an overview of the effect of the interventions for different psychiatric disorders. Second, the data was analysed to assess the collaborative care criteria (CCC) and collaborative care scores (CCS). The CCC are defined and scored (by author CK) as follows (Gunn et al. 2006):

1. A multi-professional approach to patient care: Care provided by a midwife or gynaecologist and at least one other healthcare professional (e.g. psychiatrist, psychologist, social worker, psychiatric nurse).
2. Structured management plan: Evidence-based management plan according to the NICE or Marcé guideline or national guidelines derived from these (NICE 2014; Yonkers et al. 2009).
3. Scheduled patient follow-ups: One or more telephone or in-person follow-up appointments are scheduled during or after treatment to provide specific interventions, facilitate treatment adherence or monitor symptoms or adverse effects (e.g. calls between sessions, evaluation of treatment goals, follow-up or booster sessions). Monitoring symptoms for research purposes was insufficient to meet this criterion. Also, active outreach for women who missed sessions and providing telephone sessions for women who missed a session was insufficient.
4. Enhanced interprofessional communication: Team meetings, case-conferences, individual consultation/supervision, shared medical records, patient-specific written or verbal feedback between caregivers. Communication to check whether all involved therapists work according to the same instructions was not scored.

We contacted authors for additional information on CCC, when certain information was missing or unclear (*n* = 13, 3 replies). The CCS is the total number of criteria met by an intervention, and we described the relation between the CCS and the effect of the intervention on maternal mental health. The effect of the intervention was cited as positive ( +) when maternal mental health was significantly better in (a part of) the intervention group compared to the control group at ≥ 1 follow-up moment. The score was + / − when there was not a significant difference between both groups and negative ( −) when the maternal mental health was significantly worse in the intervention group compared to the control group.

### Quality assessment

To assess the methodological quality of the included studies we used the Cochrane risk-of-bias tool for randomized trials (Higgins et al. 2011). The quality of all studies was assessed on the following domains: random sequence generation, allocation concealment, blinding of participants and personnel, blinding of outcome assessment, incomplete outcome data and selective reporting. Each item was rated as low, unclear or high risk of bias. Studies were classified as high quality (not fulfilling 0–1 criterion), moderate (not fulfilling 2–3 criteria) and low quality (not fulfilling > 3 criteria).

## Results

### Study characteristics

**Fig. 1:**
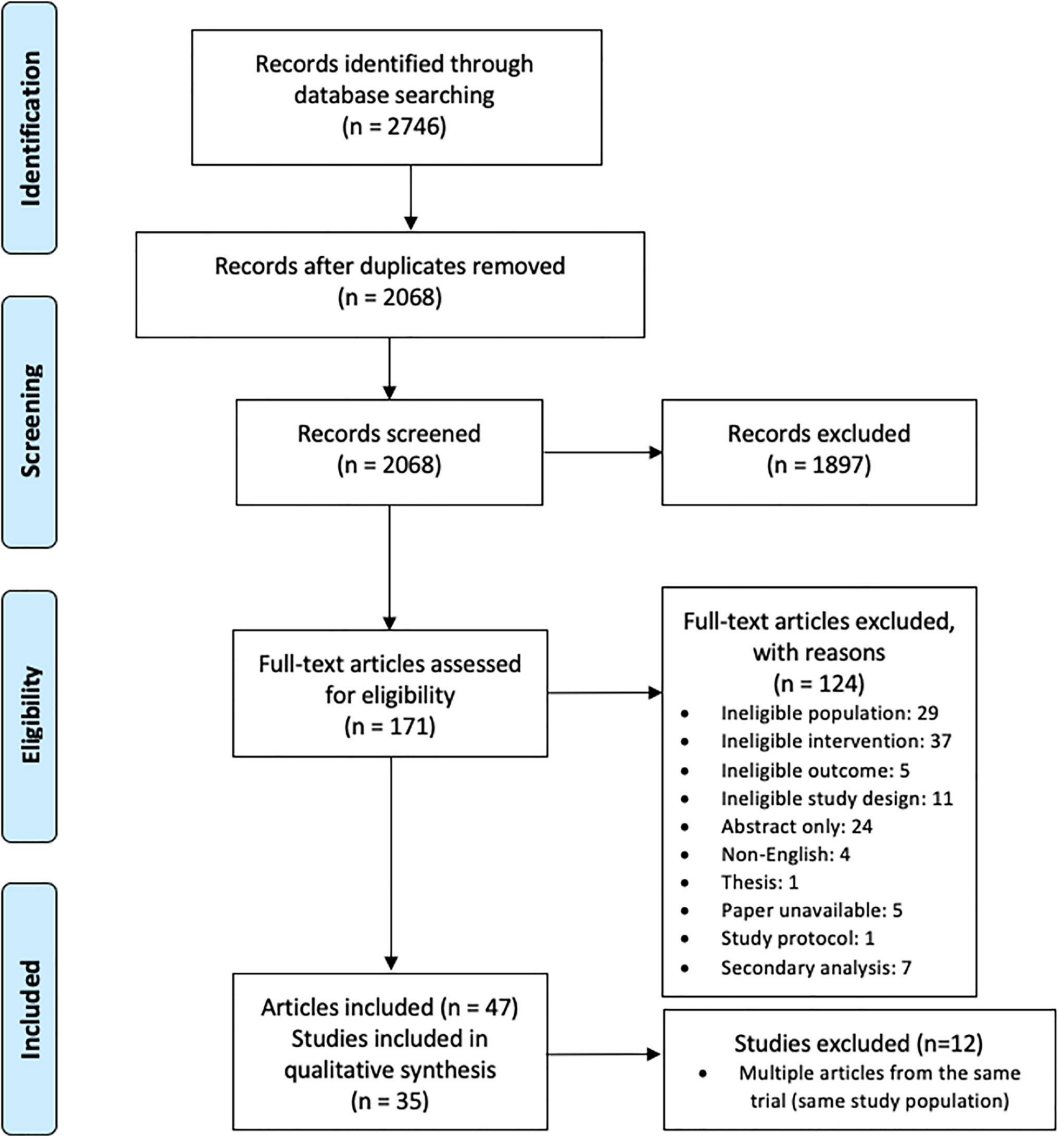
Flow diagram

Two thousand seventy hundred forty-six records were identified, of which 171 were assessed for eligibility based on title and abstract; 47 articles met the inclusion criteria (see Fig. 1 flow diagram). These articles describe 35 different multi-outcome trials, published between 2000 and 2021 (see online resource 2).

Depression was the most common studied mental disorder (*k* = 23), followed by a combination of depression and anxiety (*k* = 5), insomnia (*k* = 3) and tocophobia (*k* = 3). One study (*k* = 1) focused on a variety of psychiatric and personality disorders. Of all studies, 16 included women with a combination of psychiatric symptoms and psychosocial risk factors (e.g. low socio-economic status, deprived area). CBT (*k* = 15) and IPT (*k* = 6) were the most studied interventions. Other interventions studied more than once were psychoeducation (*k* = 4), mindfulness-based therapy (*k* = 4) and multicomponent therapy (*k* = 2). There were no studies on pharmacotherapy only. A considerable part of the interventions (*k* = 15) was provided to groups of women. The number of sessions varied between 2 to 16 sessions, which were mostly provided once a week.

### Depression and anxiety

#### Cognitive behavioural therapy (CBT)

Most included RCT’s (*k* = 12) investigated the effect of CBT on antenatal depression. Part of the studies (*k* = 6) examined individual CBT (6–14 sessions) modified to pregnant women, focusing on the specific problems these women were facing. Three pilot studies (Cho et al. 2008; Burns et al. 2013; Milgrom et al. 2015) found significantly lower levels of depressive or anxiety symptoms in the CBT group compared to the care as usual group. Of the 3 larger trials, one study (Bittner et al. 2014) found an intervention effect for women with high depressive symptoms, one study (Austin et al. 2008) described no significant effect, and one study (Burger et al. 2020) showed a negative effect of CBT compared to usual care. The other 6 studies examined CBT modified to women with additional psychosocial problems (e.g. low-income, minority group). Four studies (O'Mahen et al. 2013; Alhusen et al. 2021; Le et al. 2011; Jesse et al. 2015), including two pilot studies (O'Mahen et al. 2013; Alhusen et al. 2021), reported (significantly) lower depressive symptoms in the CBT group compared to the care as usual group. However, one of these study (Jarde et al. 2016, 2015) found a treatment effect for high-risk African American women only, and one study was not powered to test for statistical significance. The other 2 studies, (Muñoz et al. 2007) did not find a significant difference between both groups.

#### Interpersonal therapy (IPT)

Overall, 4 studies (Spinelli and Endicott 2003; Zlotnick et al. 2006; Zlotnick et al. 2016; Grote et al. 2009) concluded that IPT (4–16 sessions) was more effective than usual care or a parenting education programme. The other 2 studies (Spinelli et al. 2013; Lenze et al. 2020) did not report a significant effect. All interventions were provided to women with both psychiatric and psychosocial problems. A recently published pilot study compared IPT with CBT, which both showed a positive effect on depressive symptoms (Evans et al. 2021).

#### Psychoeducation

Both studies on psycho-education (Lara et al. 2010; Zhao et al. 2019) (6–8 sessions) showed a significantly positive effect on depression rates or symptoms compared to care as usual and the self-help book only group.

#### Mindfulness-based therapy

Three articles (Yazdanimehr et al. 2016; Zemestani, et al. 2020; Lönnberg et al. 2020) described mindfulness-based interventions (e.g. CBT, mindfulness-based childbirth and parenting group), which all showed positive findings concerning depressive and/or anxiety symptoms compared to usual care.

#### Multicomponent therapy

Two RCT’s (Grote et al. 2015; Ravesteyn et al. 2018) examined the effect of a multicomponent treatment provided by a multi-disciplinary team. An intervention^47^ providing the choice of brief IPT and/or pharmacotherapy revealed a significantly positive effect compared to care as usual. A combination of weekly CBT, psychoeducation, body-oriented and relaxation therapy^48^ was signifcantly not more effective than individual counselling sessions.

### Other psychological therapies

Both problem-solving skills training (Khamseh et al. 2019) (5 sessions) and behavioural activation (Dimidjian et al. 2017) (10 sessions) were reported as effective in reducing depressive (and anxiety) symptoms. A group preventive risk-reducing treatment to prevent postpartum depression (Brugha et al. 2000) and psychosomatic programming (Ortiz Collado et al. 2014) did not reveal a significant effect of the intervention compared to usual care.

### Insomnia

Three studies (Manber et al. 2019; Rezaei et al. 2015; Khatibi et al. 2021) showed that treating insomnia might lead to reduction of depressive symptoms. Both, 5 individual sessions of CBT and 4 sessions of group behavioural sleep education, significantly reduced depressive symptoms during pregnancy.

### Tocophobia

Two sessions of telephone psychoeducation by a midwife (Toohill et al. 2014) as well as cognitive therapy for tocophobia (Saisto et al. 2001) significantly reduced the level of fear of childbirth or birth-related concerns compared to written information about childbirth. Also, mindfulness-based childbirth and parenting group sessions (Veringa-Skiba et al. 2021) led to significantly lower levels of fear of childbirth compared to enhanced care as usual.

### Secondary outcomes

Positive effects of perinatal interventions on birth, neonatal and infant outcomes are described (e.g. reduction of preterm birth rate, lower caesarean rate, higher scores on self-regulation and stress-reactivity at 9 months) (see online resource 3).

### Collaborative care score

Table 1 shows an overview of the CCC of all studies. Of the 35 studies, 33 interventions met at least 1 criterion, and most studies met 2 criteria. A multi-professional approach to patient care was the most scored, and interprofessional communication was the least scored criterion. Of the 35 studies, 6 studies met all CCC. The studies with high CCS scores of 3 or 4 (*n* = 13) were all based on a structured management plan according to NICE or Marcé guidelines (CBT, IPT, multicomponent, psychoeducation) and provided by multiple professionals to depressive women. All interventions with a score of 4 were provided to women with additional psychosocial risk factors. Whereas the CCS 3 studies provided follow-up sessions after treatment only, the CCS 4 studies provided more often follow-up sessions during treatment. Also, more professionals were involved in the CCS 4 studies (2 to 6 professionals) than in CCS 3 studies (2 professionals). Of the 29 multi-professional interventions, 25 interventions were provided by a gynaecologist/midwife and a mental health therapist. The other 4 interventions were provided by a team of 3 to 6 different professionals. All 4 studies provided by one professional were provided by midwifes and/or obstetricians (and not by a mental health worker).

**Table 1:** The collaborative care criteria and collaborative care scores sorted by intervention/diagnosis from a high to a low CCS score

Study	Structured management plan (* = Not according to NICE/Marcé guideline)	Professional (n + gyn^a^)	Scheduled patient follow-ups	Enhanced interprofessional communication	CCS
El-Mohandes	**CBT**	1	2 booster sessions	Individual consultation, supervision	**4**
Jesse	CBT (group)	3	Weekly booster session telephone calls	Individual consultation, shared medical records	**4**
Le	CBT (psycho-educational, group)	1	3 individual booster sessions	Team meetings	**4**
Austin	CBT (group)	1	Follow-up session		3
Burger	CBT	1	Sessions up to 3 months postpartum		3
Cho	CBT	1	In the final session plans for follow-up were discussed		3
Muñoz	CBT (group)	1	4 booster sessions		3
Alhusen	CBT (group)	1			2
Bittner	CBT (group)	1			2
Burns	CBT	1			2
Milgrom	CBT	1			2
O’mahen	CBT	1			2
Yazdanimehr	CBT (mindfulness)	1			2
Zemestani	CBT (mindfulness, group)	1			2
Khatibi	CBT	Unknown			1
Lenze	**IPT**	1	Contact between sessions, postpartum sessions	Team meetings, supervision	**4**
Grote (09)	IPT	1	Maintenance sessions up to 6 months postpartum		3
Zlotnick	IPT (group)	1	Booster session		3
Evans	IPT	1			2
Spinelli (03)	IPT	1			2
Spinelli (13)	IPT	1			2
Grote (15)	**Multicomponent** (choice of IPT or antidepressant)	3	Telephone sessions, in-person visits up to 18 months	Team meetings	**4**
Van Ravesteyn	Multicomponent (group)	5	Weekly evaluation of treatment goals	Team meetings	**4**
Lara	**Psychoeducation** (group)^b^	1	2 individual booster sessions, calls between sessions		3
Zhao	*Psychoeducation (group)^b^	1	Continuous support between sessions (individual counselling via telephone or email)		2
Manber	CBT (**insomnia**)	1			2
Rezeai	Behavioural health sleep education (group, insomnia)	Unknown			1
Saisto	Cognitive therapy (**tocophobia**)	-			1
Toohill	*Telephone psychoeducation (tocophobia)	-			0
Veringa	*Mindfulness-based childbirth/parenting (group, tocophobia)	-			0
Brugha	*Preventative risk-reducing treatment (group)	1	Reunion class		2
Dimidjian	Behavioural activation	1			2
Khamseh	*Problem-solving skills training	3		Team meetings, individual consultation, verbal feedback	2
Lönnberg	*Mindfulness-based childbirth/parenting (group)	1	Reunion session		2
Ortiz	*Psychosomatic programming	-	Between sessions a follow-up phone call		1

^a^The number of different involved professionals besides a gynaecologist or midwife

^b^Psychoeducation with the provision of a manual (information, working forms, checklists) meets the criteria for facilitated self-help, described as an evidence-based management plan (NICE guideline). Without the provision of a self-help manual, psychoeducation is not described as an evidence-based management plant

### Impact collaborative care score

Studies with a CCS of 2 showed the most interventions (*k* = 14) with a positive effect on maternal mental health (see Table 2 and more extensive in online resource 4). Interventions with higher scores did not show a positive effect more often than interventions with lower scores. Of the 24 interventions with a positive effect, 20 studies met the criterion of a multi-professional approach to patient care. The retention and compliance rates are also described in Table 2. These do not show a clear pattern. However, all studies with the highest retention rates of ≥ 90% (*k* = 7) and studies with the highest compliance rates of ≥ 60% (*k* = 6) met the criterion of a multi-professional approach to patient care. The few studies that scored on interprofessional communication had predominantly high retention and compliance rates. Although a higher CCS did not always show a positive effect or higher retention and compliance rates, almost all interventions with a positive effect on maternal mental health and with high retention and compliance rates met the criterion of a multi-professional approach to patient care.

**Table 2:** The relation between the collaborative care score and the effect of the intervention on maternal mental health and retention and compliance rates; the CCC score is the total number of collaborative care criteria met by an intervention

CCC score	0	1	2	3	4
Positive effect intervention	Veringa-Skiba Toohill	Khatibi Rezeai	Alhusen Bittner Burns Dimidjian Evans Khamseh Lönnberg Manber Milgrom O’Mahen Spinelli (03) Yazdanimehr Zemestani Zhao	Cho Grote (09) Lara Zlotnick	Grote (15) Jesse Le
No difference		Ortiz Saisto	Brugha Spinelli (13)	Austin Mūnoz	El-Mohandes Lenze Ravesteyn
Negative effect intervention				Burger	
Retention rate range (%)	75	59–89	45–100	31–91	56–98
Compliance rate range (%)	21	54	23–100	49–80	43–84

### Study quality

Most studies had a medium risk of bias, due to insufficiently describing one or more criteria, leading to an unclear risk of bias (see online resource 5). Blinding of participants and personnel to the given intervention was most times impossible, which could have led to a high risk of performance bias. Additionally, most outcomes were self-reported measures, which could have attributed to the risk of bias when participants were not blinded for the intervention.

## Discussion

### Main findings

The aim of this study was to give a systematic overview of all current antenatal mental health interventions and to describe the role of collaborative care. The current available interventions are almost all either based on a structured management plan according to NICE or Marcé or multidisciplinary, which means almost all studies met at least one collaborative care criterion. Interventions with a higher collaborative care score did not more often show a positive effect on maternal mental health.

Almost all included trials (*n* = 28) focused on treating women at risk of a depressive disorder. CBT and IPT were the most studied interventions. Both therapies were predominantly effective in reducing depressive and anxiety symptoms. The effects of other interventions are based on a few trials with small numbers of participants. Potentially beneficial interventions are mindfulness-based therapy (3 trials), multicomponent treatment (2 trials), psychoeducation (2 trials) and behavioural activation (1 trial). The results of the few studies on tocophobia and insomnia showed promising findings in reducing depressive symptoms and symptoms of fear.

Although we could not find a clear effect of the CCS on maternal mental health, we did find that a multi-professional approach to patient care could be essential in the treatment of pregnant women with psychiatric and psychosocial problems. Almost all interventions with a positive effect on maternal mental health and all studies with high retention (≥ 90%) and compliance (≥ 60%) rates met this criterion. However, it was also the most common CCC, which could have had impact on its perceived prevalence in positive outcome studies. Interventions provided by a multi-professional team consisting of more than 2 different professionals (> gynaecologist/midwife and a mental health therapist) were rare, as well as interprofessional communication which was the least scored criterion.

### Strength and limitations

This review extends the literature about the challenges in antenatal mental health care (Nillni et al. 2018; Ravesteyn et al. 2017; Dennis 2005) by giving a new broad overview of current available therapies in this field and describing the role of collaborative care in this treatment. We limited our study to RCT’s only; therefore, we could have missed other potentially contributing studies. CBT and IPT were the most studied interventions, but we found a high heterogeneity within these interventions (e.g. content and number of sessions). Due to the focus on collaborative care instead of intervention and outcome protocols, the heterogeneity between studies is not fully classified in the results section. Also, the studies occurred across the world, in different healthcare systems and over a 20-year span, during which the antenatal care field, including collaborative care, was rapidly evolving. Retention and compliance rates were difficult to compare, because of the variation in the number of provided sessions and the time of follow-up. Another limitation is that the method of this study partly relied on the additional information on CCC send by authors, which could have led to an underestimation of the CCS’s because not all authors responded. Last, we included two pilot studies that were not powered to test for statistical significance.

### Interpretation

To our knowledge, this is one of the first reviews about collaborative care in the setting of antenatal mental health care. Research in antenatal mental health care mainly focuses on the effect of psychotherapy on depressive symptoms. The predominantly positive findings of CBT and IPT are in line with previous research that showed that CBT and IPT—interventions advised by the NICE guideline—are effective in treating antenatal depression (Sockol 2015; Sockol 2018; Nillni et al. 2018). The treatment of women with additional psychosocial problems, resembling clinical practice, is less studied. A systematic review of (Nillni et al. 2018) about the antenatal treatment of depression and anxiety disorders also included women with psychosocial problems. However, this review showed, unlike our review, mixed findings of both CBT and IPT in this population. Especially, the interventions with a high CCS focused on this population. This could mean that a high CCS can have a positive effect in populations with additional psychosocial problems.

As already noted, collaborative care in primary care setting is effective in improving psychiatric symptoms and adherence to treatment (Thota et al. 2012; Alhusen et al. 2021, 2012, Gunn et al. 2006). In obstetrics setting, a previous review of qualitative and quantitative studies concluded that professionals in the antenatal field are supportive of increased collaboration with other professionals because of the expected benefits for mother and child (Myors et al. 2013). Key components were identified to make this process happen, like funding and resources for collaboration, guidelines and training and education of staff. (Melville et al. 2014) conducted an RCT, in which a multidisciplinary care intervention adapted to obstetrics and gynaecology clinics was studied. The study reported a greater improvement in psychiatric symptoms, better adherence to treatment and a greater satisfaction with care than in the usual care group. Although there is limited evidence about the role of collaborative care in the antenatal mental healthcare setting, this review showed the developments in the field, because almost all interventions were provided by multiple professionals. However, interventions provided by multidisciplinary teams of more than 2 different professionals (e.g. social worker) are rare. Also, remarkable is that just a few studies met the criterion of interprofessional communication. That means that obstetric and mental healthcare professionals were most times both involved, but that communication, in a structured way, between them often did not take place or was not reported. Further research should focus on the effect of communication and collaboration between the different professionals, which will enable personalized medicine, including shared decision-making between the patient and the different involved caregivers. Both are important in (mental) health care and will eventually lead to improved treatment adherence and more meaningful outcomes for the patient (Drake et al. 2009). Because of the low number of trials that met all collaborative care criteria, more trials are needed, to make a clear conclusion about the impact of collaborative care on maternal mental health and treatment adherence.

Last, this review shows which evidence is missing in the field of antenatal mental health care. Almost all studies were about interventions provided to women with depressive symptoms. Despite the high incidence of anxiety symptoms during pregnancy, just 5 trials focused on women at risk of depression and anxiety. Only 3 trials were about women with tocophobia or insomnia. This shows there is a clear need for more trials on psychiatric disorders other than depression. Additionally, more research to interventions except from CBT and IPT could lead to positive consequences in the field. For example, promising results of mindfulness-based therapy were found in this review. Although there is raising awareness for child and partner outcomes, almost all current outcomes are short-term mother or birth outcomes. Because of the negative long-term effects of antenatal mental health disorders, for future research, it is recommended to examine long-term outcomes of the whole family system, so mother, partner and child.

## Conclusions

In conclusion, there is an important lack of evidence in the field of antenatal mental health care. Available evidence focuses on studies evaluating CBT and IPT for treating antenatal depression. More research is recommended on other psychiatric disorders, interventions and long-term follow-up outcomes. Additionally, collaborative care and especially a multi-professional approach to patient care are already implemented in most available interventions. However, interprofessional communication and multidisciplinary teams of more than two different professionals are rare. More trials are needed on the impact of interprofessional communication and on the mechanisms by which collaborative care meets antenatal mental health needs to be more conclusive whether and in which way collaborative care is a key component in antenatal mental health care.

## Supplementary Information

Below is the link to the electronic supplementary material.

Supplementary file1 (DOCX 19 KB)

Supplementary file2 (DOCX 1299 KB)

Supplementary file3 (DOCX 1283 KB)

Supplementary file4 (DOCX 25 KB)

Supplementary file5 (DOCX 1408 KB)
