# Supplementary material for: Is collaborative care a key component for treating pregnant women with psychiatric symptoms (and additional psychosocial problems)? A systematic review
Source: Arch Womens Ment Health. 2022 Sep 26;25(6):1029–39. doi: 10.1007/s00737-022-01251-7 (PMC9734206; doi:10.1007/s00737-022-01251-7)
Supplement: Supplementary file 1 — Supplementary file1 (DOCX 19 KB) [file 737_2022_1251_MOESM1_ESM.docx]

| Pregnancy AND  Gestation  Pregnant women  Gravidity  Childbearing  Prenatal period  Antenatal period  Perinatal period  Peripartum period | Mental disorders OR  Psychopathology  Psychiatric diagnosis  Mentally ill persons  Maternal distress   1. Mood disorders or affective disorders (depressive disorders, depression) 2. Bipolar disorders 3. Schizophrenia spectrum and other psychotic disorders (psychotic disorders, schizophrenia) 4. Personality disorders 5. Adjustment disorders 6. Anxiety disorders or anxiety (agoraphobia, obsessive-compulsive disorder, panic disorder, phobic disorder) 7. Traumatic stress disorders 8. Feeding and eating disorder (anorexia nervosa, bulimia nervosa) | Psychosocial problems AND  Vulnerable population  Social isolation  Psychosocial deprivation  Complex social situations | Psychiatry/therapy  Psychiatry/pharmacology  Pharmacotherapy  Psychopharmacology  Drug therapy:   1. Antipsychotic agents 2. Tranquilizing agents 3. Anti-anxiety agents, anxiolytics 4. Antimanic agents 5. Psychotropic drugs, neuroleptics, anticonvulsants 6. Antidepressive agents, antidepressants 7. Mood stabilizers   Cognitive behavioural therapy  Psychotherapy  Psychoeducation  Psychiatric social work  Integrated care  Mental health services  Maternal health services  Prenatal care  Perinatal care  Antenatal care  Ambulatory care  Collaborative care |
| --- | --- | --- | --- |

Table S1 Search terms

**Is collaborative care a key component for treating pregnant women with psychiatric symptoms (and additional psychosocial problems)? A systematic review.** Celine K. Klatter, Leontien M. van Ravesteyn, Jelle Stekelenburg

Archives of Women’s Mental Health

Corresponding author:

C.K. Klatter

University of Groningen

Email: [celine.klatter@mcl.nl](mailto:celine.klatter@mcl.nl)
