# Supplementary material for: Is collaborative care a key component for treating pregnant women with psychiatric symptoms (and additional psychosocial problems)? A systematic review
Source: Arch Womens Ment Health. 2022 Sep 26;25(6):1029–39. doi: 10.1007/s00737-022-01251-7 (PMC9734206; doi:10.1007/s00737-022-01251-7)
Supplement: Supplementary file 2 — Supplementary file2 (DOCX 1299 KB) [file 737_2022_1251_MOESM2_ESM.docx]

| Study | Country,  Setting | Participants  (n) | Intervention,  Duration | Comparison | Outcome measures | Outcome of psychiatric symptoms | RR (%)  CR (%) |
| --- | --- | --- | --- | --- | --- | --- | --- |
| **Alhusen (2020)** | United States (Maryland), obstetrical clinics, a predominantly African American population with a low socioeconomic status | Pregnant women exhibiting moderate to severe depressive symptomatology (EPDS >12)  (60) | CBT group treatment,  6 weekly 2-hour sessions (Mothers and Babies Course, Muñoz (2007)),  referral to a social worker | Usual care, referral to a social worker | EPDS | Greater decrease in depressive symptomatology from baseline to 12 weeks postpartum in the intervention group compared to the control group (not powered to test for statistical significance) | 93  70 |
| **Austin**  **(2008)** | Australia,  Antenatal clinic at the Royal Hospital for Women | Pregnant women with an EPDS score of >10 and/or a score of >23 on the ANRQ, or a reported prior history of depression  (277) | CBT group treatment,  6 weekly 2-hour sessions | Information booklet | EPDS | No significant differences between groups at post-intervention, 2 months, and 4 months postpartum | 60 |
| **Bittner**  **(2014)^a^** | Germany,  Private gynaecologist practices | Medically verified pregnant women and screening results of at least 1 questionnaire above study cut-off (PDQ >14, STAI >36, BDI-V >20)  (160) | CBT group treatment,  8 sessions of 90 minutes | Usual care | EPDS | Participants with high depressive symptoms (EPDS >10) had significantly lower depressive symptoms in the intervention group compared to the control group at 3 months postpartum | 45  26* |
| **Brugha (2000)** | United Kingdom, General hospital antenatal clinics | Primiparous women who were identified, by screening (GHQ-D), to be at increased risk of postnatal depression  (209) | Preventative risk-reducing group treatment,  6 weekly 2-hour session | Usual care | EPDS | No significant differences between groups at 3 months postpartum | 91 |
| **Burger (2019)** | The Netherlands, Midwifery practices and hospitals | Pregnant women with at least moderate anxiety or depression (STAI >42 or EPDS >12)  (282) | CBT,  10-14 sessions (6-10 during pregnancy) from 20 weeks’ gestation up to 3 months postpartum | Usual care | EPDS  STAI | Significantly higher depressive and anxiety symptoms in the intervention group compared to the control group at 24 weeks’ gestation  No significant differences from 36 weeks’ gestation till 18 months postpartum | 67  49 |
| **Burns (2013)^d^** | United Kingdom, Midwives in North Bristol, a mainly urban setting with some areas of high deprivation | Pregnant women who screened positive on a 3-question depression screen (used routinely by midwives) and met ICD-10 criteria for depression assessed using the CIS-R  (36) | CBT,  12 sessions at the woman’s home | Usual care | EPDS | Significantly lower depressive symptoms in the intervention group compared to the control group at 15- and 33-weeks post-randomisation | 89  56 |
| **Cho (2008)** | South Korea,  6 obstetrics and gynaecology clinics (at a University Medical centre and at private clinics) | Pregnant women who scored more than 16 points on the BDI and met DSM-IV criteria for a depressive disorder (SCID-I)  (27) | CBT,  9 bi-weekly 1-hour sessions | Psychoeducation,  1 session | BDI | Significantly lower depressive symptoms in the intervention group compared to the control group at 1 month postpartum | 80  80 |
| **Dimidjian (2017)** | United States (Washington, Minnesota, Colorado, Georgia), Integrated healthcare systems providing general medicine and mental health care, predominantly women on Medicaid or low-income programs | Pregnant women with a baseline score of >10 on the PHQ-9  (163) | Behavioural activation,  10 sessions | Usual care | PHQ-9  GAD-7 | Significantly lower depressive and anxiety symptoms in the intervention group compared to the control group at 5- and 10-weeks follow-up and 3 months postpartum | 84 |
| **El-Mohandes(2008)^b^** | United States (Washington), Prenatal care clinics | African American or Latino pregnant women who had at least 1 of 4 risk factors of interest (active smoking, environmental tobacco smoke exposure (SFF), depression (BDI), or intimate partner violence (AAS))  (1044) | Integrated cognitive behavioural intervention to reduce behavioural and psychosocial risks,  4-8 sessions | Usual care | HSCL-D | No significant differences between groups 10 weeks postpartum | 77 |
| **Evans (2021)** | United Kingdom, National Health Service psychological treatment services | Pregnant women with an EPDS score of >10 and an ICD-10 mild or moderate depression determined by the CIS-R  (52) | Interpersonal counselling, a brief low-intensity form of IPT, 6 30-45-minute sessions | Low-intensity perinatal- specific CBT, 6 individual 30-45-minute sessions | EPDS | Both groups reported an improvement in depressive symptoms at 12 weeks post-randomization compared to baseline, the mean drop in EPDS score was 4.4 (sd. 5.1) for the intervention group and 4.0 (sd. 4.8) for the control group (not powered to test for statistical significance) | 81  *46 |
| **Grote (2009)** | United States (Pennsylvania),  Public care outpatient obstetrics and gynaecology clinic of a large women’s hospital | Pregnant women meeting criteria for depression on the EPDS (score >12)  (53) | Brief IPT made culturally relevant to socioeconomically disadvantaged women,  8 acute IPT-B sessions before birth and maintenance IPT (biweekly or monthly) up to 6 months postpartum | Enhanced usual care, depression education materials and referral to the behavioural health centre | EPDS | Significant lower depressive symptoms in the intervention group compared to the control group 3 months post-baseline and 6 months postpartum | 88  68* |
| **Grote (2015)** | United States (Washington),  A 10-site public health system | Pregnant women with a diagnosis of probable MDD (at least 5 symptoms scored as >2 with one cardinal symptom on the PHQ-9, plus a functional impairment item) or a diagnosis of probable dysthymia based on the MINI  (168) | MOMCARE, a culturally relevant, collaborative care intervention providing choice of brief IPT of 8 sessions and/or pharmacotherapy | MSS-Plus only, usual multi-disciplinary care for women on Medicaid | SCL-20 | Significantly lower depressive symptoms in the intervention group compared to the control group at 6 months and 18 months post-baseline | 96  84 |
| **Jesse (2015)** | United States (Southeastern),  Local health department prenatal clinic, affiliated regional perinatal centre | Pregnant women with low-moderate or high risk for antepartum depression (EPDS >4) and who are enrolled in Medicaid or are low-income based on Special Supplemental Nutrition Program for Women, Infants, and Children  (146) | CBT group treatment, a culturally tailored intervention adapted for rural and minority pregnant low-income women,  6-weekly 2-hour sessions | Usual care | EPDS | Significant lower depressive symptoms for the high-risk African American women in the intervention group compared to the control group post-intervention and at 1-month follow-up | 56  54 |
| **Khamseh (2019)** | Iran,  Prenatal care clinics of a city hospital | Pregnant women having mild-to-severe depression (score of 11 or higher) according to the BDI  (70) | Problem-solving skills training,  5 weekly 90-minute sessions | Usual care | BDI | Significant lower depressive symptoms in the intervention group compared to the control group post-intervention and at 1 month follow-up | 100  100 |
| **Khatibi (2021)** | Iran,  Comprehensive urban healthcare centres | Primiparous pregnant women having sleep quality score more than 5 based on the PSQI  (56) | Cognitive behavioural counselling, 5 weekly 90-minute sessions | Usual care | DASS-21  PSQI | Significantly better sleep quality scores and lower depressive symptoms in the intervention group compared to the control group immediately post-intervention and at 8 weeks follow-up | 89 |
| **Lara (2010)** | Mexico,  Hospital, women’s clinic and a community health care centre | Low-income pregnant women at risk of depression based on a score of 16 or higher on the CES-D or a self-reported history of depression  (377) | Psycho-educational group intervention,  8 weekly 2-hour sessions | Usual care plus self-help book on depression | BDI  Incidence MD | No significant differences between groups at 6 weeks and 4-6 months postpartum  The cumulative incidence of major depression over three time periods was significantly lower in the intervention (10,7%) than the control group (25%) | 31 |
| **Le (2011)** | United States (Washington),  A community-based health centre and a hospital clinic | Latino pregnant women at high risk of depression defined as scoring 16 or higher on the CES-D or with a self-reported personal or family history of depression  (217) | Psycho-educational CBT group treatment (Mothers and Babies Course, Muñoz (2007)), 8 weekly 2-hour sessions plus 3 individual booster sessions 6 weeks, 4 and 12 months postpartum | Usual care | BDI | Significantly lower depressive symptoms in the intervention group compared to the control group immediately post-intervention  No significant differences between groups postpartum | 69 |
| **Lenze (2020)** | United States (Washington),  Urban prenatal clinic, (low income population, experiencing high psychosocial adversity | Pregnant women scoring >10 on the EDS and meeting depressive disorder or dysthymia criteria (SCID-IV)  (42) | (Dyadic) interpersonal psychotherapy (Grote et al., 2004; 2009) plus free diapers at each session, reminder calls, follow up calls and check-in calls,  8-weekly individual IPT sessions and at least 4-weekly postpartum sessions (dyadic component) | Enhanced treatment as usual | EDS | No significant differences between groups at 37-39 weeks’ gestation and at 3-, 6-, 9- and 12-months postpartum | 71  43* |
| **Lönnberg (2020)** | Sweden,  Maternity health clinics | Pregnant women scoring 6 or higher on the 4-item Perceived Stress Scale, having previously sought health care for mental health problems, having previous experience of depression or anxiety, or scoring 6 or higher on 3 selected items from the Childhood Trauma Questionnaire  (193) | Mindfulness-based childbirth and parenting group program,  8 2-hour sessions | Lamaze childbirth class,  3 group meetings of 3 hours | EPDS | Significantly lower depressive symptoms in the intervention group compared to the control group post-intervention | 78 |
| **Manber (2019)** | United States (California),  University-based obstetric clinics and county hospital-based obstetric clinics | Pregnant women who met DSM-V (minimum duration criterion DSM-IV) criteria for insomnia disorder  (194) | CBT for insomnia,  5 individual sessions | Control intervention,  5 individual sessions | EPDS | Significantly lower depressive symptoms in the intervention group compared to the control group post-intervention | 74  74 |
| **Milgrom (2015)^c^** | Australia,  Two general hospitals and services at the public and private sector | Pregnant women with a score of >13 on the EPDS and a DSM-IV diagnosis (SCID) of a depressive disorder  (54) | CBT,  8-weekly one-hour sessions | Usual care | BDI  BAI | No significant differences between groups post-treatment and 9 months postpartum  Significantly lower anxiety scores post-treatment in the intervention group compared to the control group at 9 months postpartum | 85  63 |
| **Muñoz (2007)^e^** | United States (California),  A public sector women’s clinic, predominantly low-income Latina women | Pregnant women at high risk of depression (a history of MDE and/or a score of 16 or higher on the CES-D)  (45) | Cognitive-behavioural mood management (Mothers and Babies Course)  12-weekly group sessions and 4 booster sessions (1,3,6 and 12 months postpartum) | Usual care | EPDS | No significant differences between groups post-intervention and 1, 3, 6 and 12 months postpartum | 91 |
| **O’mahen (2013)** | United States (Michigan),  Obstetric clinics in urban settings, predominantly low income and undeserved women | Pregnant women meeting DSM-IV criteria for Major Depressive Disorder (MDD)  (55) | CBT modified to address the needs of perinatal, low-income women with major depressive disorder,  12 50-min sessions | Usual care | BDI | Significantly lower depressive symptoms in the intervention group compared to the control group at 16-week post-randomization and 3-month follow-up | 70  23 |
| **Ortiz (2014)** | Spain and France, Public hospitals | Pregnant women (and their partners) identified at middle or low socioeconomic status and moderate to high risk of PPD (interview)  (184) | Psychosomatic programming,  10 2-hour sessions | Usual care | EPDS | No significant differences between groups | 75 |
| **Rezeai (2015)** | Iran,  Health-elected city centre shuttle | Pregnant women with insomnia or poor sleep quality (PSQI >5 and BDI >11)  (96) | Group behavioural health sleep education,  4 1-hour sessions with one-week interval | Usual care | BDI | Significantly lower depressive symptoms in the intervention group compared to the control group at 1- and 2-months post-intervention | 88 |
| **Saisto (2001)** | Finland,  Outpatient clinic of the department of Obstetrics and Gynaecology at a University Hospital | Pregnant women with fear of vaginal delivery, as diagnosed by a specific questionnaire (five or more affirmative answers or request for caesarean)  (176) | Intensive therapy for fear of childbirth,  Mean 3.8 +1.0 sessions | Conventional therapy,  Mean 2.0 + 0.6 sessions | BDI | No significant differences between groups during pregnancy and 3 months postpartum | 67 |
| **Spinelli (2003)** | United States (New York),  Outpatient clinics, prenatal clinic in the Department of Obstetrics and Gynaecology, community outreach programs, predominantly Hispanic women with few support systems and low socioeconomic status | Pregnant women who met DSM-IV criteria for major depressive disorder and scored 12 or higher on the HDRS  (50) | Interpersonal psychotherapy,  16-weekly 45-minute sessions | Parenting education program,  16-weekly 45-minute sessions (therapist-led) | EPDS | Significantly lower depressive symptoms in the intervention group compared to the control group over the course of treatment | 84 |
| **Spinelli (2013)** | United States (New York),  Obstetric departments of different hospitals in New York | Women who met DMS-IV criteria for major depressive disorder and scored 12 or higher on the HDRS-17  (142) | Interpersonal psychotherapy,  12-weekly 45-minute sessions | Parenting education program,  12-weekly 45-minute sessions | EPDS | No significant differences between groups at the 4^th^, 8^th^ and 12^th^ week of treatment | 69 |
| **Toohill, (2014)^h^** | Australia,  Antenatal clinics of three hospitals | Pregnant women reporting high fear of childbirth (defined as a score of >66 on the W-DEQ A)  (339) | Telephone psycho-education counselling intervention,  2 sessions at 24 and 34 weeks of pregnancy with a mean duration of respectively 58 and 45 minutes | Decision-aid booklet on childbirth choices | EPDS  WDEQ-A | No significant differences between groups at 36 weeks’ gestation and at 6 weeks postpartum  Significantly lower level of fear of childbirth at 36 weeks’ gestation in the intervention group compared to the control group | 59  54 |
| **Van Ravesteyn (2018)** | The Netherlands,  A clinic for perinatal psychiatry at a university hospital, socioeconomically deprived neighbourhoods, multi-ethnic population | Pregnant women with a psychiatric and/or personality disorder verified with the SCID by a trained medical doctor  (158) | Group-based multicomponent treatment comprising CBT, psychoeducation, body-oriented and relaxation therapy,  Weekly, all-day (6 hours), median number of 4 sessions (range 1-23) | Counselling sessions,  Weekly or monthly, median number of 4 sessions (range 1-23) | EDS | No significant differences between groups at 6 weeks postpartum | 98 |
| **Veringa-Skiba (2021)** | The Netherlands,  Midwifery care settings | Pregnant women experiencing a high fear of childbirth (W-DEQ-A >66 and self-confirmed fear of childbirth)  (141) | Mindfulness-based childbirth and parenting (MBCP), 9 weekly 3-hour group sessions | Enhanced care as usual (ECAU), 2 individual 90-minute sessions | W-DEQ-A | Significantly lower symptoms of fear of childbirth in the MBCP group compared to the ECAU group postintervention (26-36 weeks’ pregnancy) | 75  21 |
| **Yazdanimehr (2016)** | Iran,  Health care centres | Pregnant women scoring >13 on the EDS and >16 on the BAI  (80) | Mindfulness-integrated cognitive behaviour therapy,  8 weekly 90-minute sessions | Usual care | EPDS  BAI | Significantly lower symptoms of anxiety and depression in the intervention group compared to the control group post-intervention and 1 month follow-up | 75 |
| **Zemestani (2019)** | Iran,  Medical and obstetric services | Pregnant women meeting DSM-V criteria for depression and anxiety disorders and scoring >20 on the BDI-II and >22 on the BAI  (38) | Mindfulness-based cognitive group therapy,  8 weekly 2-hour group sessions | Usual care | BDI  BAI | Significantly lower depressive and anxiety symptoms in the intervention group compared to the control group from baseline to post-treatment and 1 month follow-up | 79 |
| **Zhao (2019)^f^** | China,  Antenatal clinic of a major teaching hospital | Pregnant women with a medically defined obstetric complication as defined by the High-Risk Pregnancy Scoring in Shanghai and with an EPDS >9 or a PDSS >60  (352) | Psycho-educational group program,  6-weekly 90-minute sessions | Usual care | EPDS | Significantly lower depressive symptoms in the intervention group compared to the control group in late pregnancy, 3 days and 42 days postpartum | 95  39 |
| **Zlotnick (2016)^g^** | United Stated (Rhode Island),  Three prenatal clinics (a university-affiliated hospital and two primary care sites) | Pregnant women scoring >27 on the CSQ (threshold for high-risk status for PPD) and who were on public assistance  (205) | IPT-based group intervention,  4-weekly 90-minute sessions | Usual care | PPD rate | The overall depression rate in the intervention group was significantly lower than in the control group at 6 months postpartum | 58 |

Table S2 Summary table of the studies included in the qualitative synthesis

^a-i^ Additional studies on the same multi-outcome trials, but with a different author and outcome of which the outcomes of 6 studies are described in table S3 (birth, neonatal and infant outcomes): a. Richter (2014) b. Subramanian (2011), El-Mohandes (2011), Joseph (2009) c. Bleker (2019) d. Netsi (2015), Pearson (2013) e. Urizar (2011) f. Zhao (2017) g. Zlotnick (2006) h. Fenwick (2015) i. Lenze (2017).
*Women who attended > 75% of all sessions instead of 100% of all sessions (in case of missing data about the 100% compliance rate)

**Is collaborative care a key component for treating pregnant women with psychiatric symptoms (and additional psychosocial problems)? A systematic review.** Celine K. Klatter, Leontien M. van Ravesteyn, Jelle Stekelenburg

Archives of Women’s Mental Health

Corresponding author:

C.K. Klatter

University of Groningen

Email: [celine.klatter@mcl.nl](mailto:celine.klatter@mcl.nl)
