# Supplementary material for: Is collaborative care a key component for treating pregnant women with psychiatric symptoms (and additional psychosocial problems)? A systematic review
Source: Arch Womens Ment Health. 2022 Sep 26;25(6):1029–39. doi: 10.1007/s00737-022-01251-7 (PMC9734206; doi:10.1007/s00737-022-01251-7)
Supplement: Supplementary file 3 — Supplementary file3 (DOCX 1283 KB) [file 737_2022_1251_MOESM3_ESM.docx]

| Study | Country,  Setting | Participants  (n) | Intervention,  Duration | Comparison | Outcome measures | Neonatal and infant outcomes |
| --- | --- | --- | --- | --- | --- | --- |
| **Burger (2019)** | The Netherlands, Midwifery practices and hospitals | Pregnant women with at least moderate anxiety or depression (STAI >42 or EPDS >12)  (282) | CBT,  10-14 sessions (6-10 during pregnancy) from 20 weeks’ gestation up to 3 months postpartum | Usual care | Child behavioral and emotional problems (CBCL), neonatal outcomes (birth weight, gestational age at delivery, Apgar scores), Child cognitive and motor development (BSID-III) at 18 months | Women with anxiety disorders showed significantly lower infant gestational age at delivery in the CBT compared to the control group |
| **El-Mohandes (2011)**  **Subramanian (2011)** | United States, Prenatal care clinics | African American or Latino pregnant women who had at least 1 of 4 risk factors of interest (active smoking, environmental tobacco smoke exposure (SFF), depression (BDI), or intimate partner violence (AAS))  (1044) | Integrated cognitive behavioural intervention to reduce behavioural and psychosocial risks,  4-8 sessions | Usual care | Poor pregnancy outcomes ((very) low birth weight and (very) preterm birth)  Pregnancy outcomes (miscarriage, live birth, perinatal death), preterm labor, caesarean section, sexually transmitted infection, preterm birth, low birth weight, small for gestational age, NICU admission and >2 days hospitalization | The incidence of VPTB was significantly lower in the CBT compared to the control group  No significant differences between groups |
| **Lenze (2020)** | United States,  Urban prenatal clinic  (low income population, experiencing high psychosocial adversity) | Pregnant women scoring >10 on the EDS and meeting depressive disorder or dysthymia criteria (SCID-IV)  (42) | (Dyadic) interpersonal psychotherapy (Grote et al., 2004; 2009) plus free diapers at each session, reminder calls, follow up calls and check-in calls,  8-weekly individual IPT sessions and at least 4-weekly postpartum sessions (dyadic component) | Enhanced treatment as usual | Infant-Toddler Social and Emotional Assessment (ITSEA), Infant Behavior Questionnaire – Revised Very Short Form | There were minimal (no significant) differences between groups on infant temperament and infant social/emotional development |
| **Milgrom (2015)**  **Bleker (2019)** | Australia,  Two general hospitals and services at the public and private sector | Pregnant women with a score of >13 on the EPDS and a DSM-IV diagnosis (SCID) of a depressive disorder  (54)  (23) | CBT,  8-weekly one-hour sessions | Usual care | Nine-month infant outcomes (ASQ-3, ASQ-SE, IBQ-R)  Children’s epigenetic profiles (DNA-methylation) | Nine-month infant outcomes were significantly better in the intervention compared to the control group on the following domains: self-regulation, communication, high-intensity pleasure, falling recovery and negative affectivity  No significant differences between groups at 3-7-year |
| **Ortiz (2014)** | Spain and France, Public hospitals | Pregnant women (and their partners) identified at middle or low socioeconomic status and moderate to high risk of PPD (interview)  (184) | Psychosomatic programming,  10 2-hour sessions | Usual care | Preterm childbirth (<37 weeks), birth weight | The incidence of premature childbirth was significantly lower in the intervention group compared to the control group |
| **Pearson (2013)**  **Netsi (2015)** | United Kingdom, Midwives in North Bristol, a mainly urban setting with some areas of high deprivation | Pregnant women who screened positive on a 3-question depression screen (used routinely by midwives) and met ICD-10 criteria for depression assessed using the CIS-R  (24)  (25) | CBT,  12 individual sessions at the woman’s home | Usual care | Attentional bias for infant distress  Infant sleep duration and temperament at two months postpartum | The attentional biases of women who received CBT improved and became comparable to those of non-depressed women post-intervention  No significant differences between groups |
| **Veringa-Skiba (2021)** | The Netherlands,  Midwifery care settings | Pregnant women experiencing a high fear of childbirth (W-DEQ-A >66 and self-confirmed fear of childbirth)  (141) | Mindfulness-based childbirth and parenting (MBCP), 9 weekly 3-hour group sessions | Enhanced care as usual (ECAU), 2 individual 90-minute sessions | Unmedicated childbirth (birth without obstetric intervention), 1- and 5-minute newborn’s Apgar score | The intervention group had significantly more unmedicated births and a higher 1-min APGAR score compares to the control group. No significant differences between in groups in the 5-minute APGAR score |
| **Zhao (2019)** | China,  Antenatal clinic of a major teaching hospital | Pregnant women with a medically defined obstetric complication as defined by the High-Risk Pregnancy Scoring in Shanghai and with an EPDS >9 or a PDSS >60  (352) | Psycho-educational group program,  6-weekly 90-minute sessions | Usual care | Birth outcomes (gestational age of delivery, delivery mode, delivery of placenta, fetal gender, birth weight and reasons for cesarean) | The intervention group had significantly lower cesarean rate and a shorter third stage of labor |

Table S3 Summary table birth, neonatal and infant outcomes

**Is collaborative care a key component for treating pregnant women with psychiatric symptoms (and additional psychosocial problems)? A systematic review.** Celine K. Klatter, Leontien M. van Ravesteyn, Jelle Stekelenburg

Archives of Women’s Mental Health

Corresponding author:

C.K. Klatter

University of Groningen

Email: [celine.klatter@mcl.nl](mailto:celine.klatter@mcl.nl)
