# Supplementary material for: Is collaborative care a key component for treating pregnant women with psychiatric symptoms (and additional psychosocial problems)? A systematic review
Source: Arch Womens Ment Health. 2022 Sep 26;25(6):1029–39. doi: 10.1007/s00737-022-01251-7 (PMC9734206; doi:10.1007/s00737-022-01251-7)
Supplement: Supplementary file 4 — Supplementary file4 (DOCX 25 KB) [file 737_2022_1251_MOESM4_ESM.docx]

|  | CCC 1  Multiprofessional approach | CCC 2  Structured plan | CCC 3  Scheduled follow-up | CCC 4  Interprofessional communication | CCS | Maternal mental health | Retention rate | Compliance rate |
| --- | --- | --- | --- | --- | --- | --- | --- | --- |
| Grote (15) | **√** | **√** | **√** | **√** | 4 | + | 96 | 84 |
| Le | **√** | **√** | **√** | **√** | 4 | + | 69 | - |
| Jesse | **√** | **√** | **√** | **√** | 4 | + | 56 | 54 |
| Grote (09) | **√** | **√** | **√** | X | 3 | + | 88 | 68 |
| Cho | **√** | **√** | **√** | X | 3 | + | 80 | 80 |
| Zlotnick | **√** | **√** | **√** | X | 3 | + | 58 | - |
| Lara | **√** | **√** | **√** | X | 3 | + | 31 | - |
| Khamseh | **√** | X | X | **√** | 2 | + | 100 | 100 |
| Zhao | **√** | X | **√** | X | 2 | + | 95 | 39 |
| Alhusen | **√** | **√** | X | X | 2 | + | 93 | 70 |
| Burns | **√** | **√** | X | X | 2 | + | 89 | 56 |
| Milgrom | **√** | **√** | X | X | 2 | + | 85 | 63 |
| Dimidjian | **√** | **√** | X | X | 2 | + | 84 | - |
| Spinneli (03) | **√** | **√** | X | X | 2 | + | 84 | - |
| Evans | **√** | **√** | X | X | 2 | + | 81 | 46 |
| Zemestani | **√** | **√** | X | X | 2 | + | 79 | - |
| Lönnberg | **√** | X | **√** | X | 2 | + | 78 | - |
| Yazdanimehr | **√** | **√** | X | X | 2 | + | 75 | - |
| Manber | **√** | **√** | X | X | 2 | + | 74 | 74 |
| O’Mahen | **√** | **√** | X | X | 2 | + | 70 | 23 |
| Bittner | **√** | **√** | X | X | 2 | + | 45 | 26 |
| Khatibi | - | **√** | X | X | 1 | + | 89 | - |
| Rezeai | - | **√** | X | X | 1 | + | 88 | - |
| Veringa-Skiba | X | X | X | X | 0 | + | 75 | 21 |
| Toohill | X | X | X | X | 0 | + | 59 | 54 |
| Ravesteyn | **√** | **√** | **√** | **√** | 4 | +/- | 98 | - |
| El-Mohandes | **√** | **√** | **√** | **√** | 4 | +/- | 77 | - |
| Lenze | **√** | **√** | **√** | **√** | 4 | +/- | 71 | 43 |
| Mūnoz | **√** | **√** | **√** | X | 3 | +/- | 91 | - |
| Austin | **√** | **√** | **√** | X | 3 | +/- | 60 | - |
| Brugha | **√** | X | **√** | X | 2 | +/- | 91 | - |
| Spinelli (13) | **√** | **√** | X | X | 2 | +/- | 69 | - |
| Ortiz | X | X | **√** | X | 1 | +/- | 75 | - |
| Saisto | X | **√** | X | X | 1 | +/- | 67 | - |
| Burger | **√** | **√** | **√** | X | 3 | - | 67 | 49 |

Table S4 CCC and outcomes, √ meeting criterion, X not meeting criterion

**Is collaborative care a key component for treating pregnant women with psychiatric symptoms (and additional psychosocial problems)? A systematic review.**

Celine K. Klatter, Leontien M. van Ravesteyn, Jelle Stekelenburg

Archives of Women’s Mental Health

Corresponding author:

C.K. Klatter

University of Groningen

Email: [celine.klatter@mcl.nl](mailto:celine.klatter@mcl.nl)
