# Supplementary material for: Is collaborative care a key component for treating pregnant women with psychiatric symptoms (and additional psychosocial problems)? A systematic review
Source: Arch Womens Ment Health. 2022 Sep 26;25(6):1029–39. doi: 10.1007/s00737-022-01251-7 (PMC9734206; doi:10.1007/s00737-022-01251-7)
Supplement: Supplementary file 5 — Supplementary file5 (DOCX 1408 KB) [file 737_2022_1251_MOESM5_ESM.docx]

|  | Random sequence generation | Allocation concealment | Blinding of participants and personnel | Blinding of outcome assessment | Incomplete outcome data | Selective reporting | Other sources of bias | Overall risk of bias |
| --- | --- | --- | --- | --- | --- | --- | --- | --- |
| Alhusen, 2020 |  |  |  |  |  |  |  |  |
| Austin, 2008 |  |  |  |  |  |  |  |  |
| Bittner, 2014 |  |  |  |  |  |  |  |  |
| Brugha, 2000 |  |  |  |  |  |  |  |  |
| Burger, 2019 |  |  |  |  |  |  |  |  |
| Burns, 2013 |  |  |  |  |  |  |  |  |
| Cho, 2008 |  |  |  |  |  |  |  |  |
| Dimidjian, 2017 |  |  |  |  |  |  |  |  |
| El-Mohandes, 2008 |  |  |  |  |  |  |  |  |
| Evans, 2021 |  |  |  |  |  |  |  |  |
| Grote, 2009 |  |  |  |  |  |  |  |  |
| Grote, 2015 |  |  |  |  |  |  |  |  |
| Jesse, 2015 |  |  |  |  |  |  |  |  |
| Khamseh, 2019 |  |  |  |  |  |  |  |  |
| Khatibi, 2021 |  |  |  |  |  |  |  |  |
| Lara, 2010 |  |  |  |  |  |  |  |  |
| Le, 2011 |  |  |  |  |  |  |  |  |
| Lenze, 2020 |  |  |  |  |  |  |  |  |
| Lönnberg, 2020 |  |  |  |  |  |  |  |  |
| Manber, 2019 |  |  |  |  |  |  |  |  |
| Milgrom, 2015 |  |  |  |  |  |  |  |  |
| Muñoz, 2007 |  |  |  |  |  |  |  |  |
| O’Mahen, 2013 |  |  |  |  |  |  |  |  |
| Ortiz, 2014 |  |  |  |  |  |  |  |  |
| Rezaei, 2015 |  |  |  |  |  |  |  |  |
| Saisto, 2001 |  |  |  |  |  |  |  |  |
| Spinelli, 2003 |  |  |  |  |  |  |  |  |
| Spinelli, 2013 |  |  |  |  |  |  |  |  |
| Toohill, 2014 |  |  |  |  |  |  |  |  |
| Van Ravesteyn, 2018 |  |  |  |  |  |  |  |  |
| Veringa-Skiba, 2021 |  |  |  |  |  |  |  |  |
| Yazdanimehr, 2016 |  |  |  |  |  |  |  |  |
| Zemestani, 2019 |  |  |  |  |  |  |  |  |
| Zhao, 2019 |  |  |  |  |  |  |  |  |
| Zlotnick, 2016 |  |  |  |  |  |  |  |  |

Table S5 Risk of bias, low risk of bias, medium/unclear risk of bias, high risk of bias. Studies are classified as high quality (not fulfilling 0 to 1 criterion), moderate (not fulfilling 2 to 3 criteria), and low quality (not fulfilling >3 criteria). Low risk of bias: no bias detected. Medium/unclear risk of bias: therey may be risk of bias, but it is not described in sufficient detail or there is insufficient evidence that the detected problem will introduce bias. High risk of bias: bias detected.

Lo

**Is collaborative care a key component for treating pregnant women with psychiatric symptoms (and additional psychosocial problems)? A systematic review.** Celine K. Klatter, Leontien M. van Ravesteyn, Jelle Stekelenburg

Archives of Women’s Mental Health

Corresponding author:

C.K. Klatter

University of Groningen

Email: [celine.klatter@mcl.nl](mailto:celine.klatter@mcl.nl)
